# Workplace spirituality as a predictor of nursing self-efficacy and job embeddedness

**DOI:** 10.1186/s12912-026-04393-6

**Published:** 2026-02-26

**Authors:** Maysa Fekry Ahmed, Eman Sameh AbdElhay, Asmaa Moustafa Abd-ElGhani

**Affiliations:** 1 Assistant professor of Nursing Administration, Faculty of Nursing, Mansoura University, Mansoura City, Egypt; 2 Assistant professor of Psychiatric Nursing and Mental Health, Faculty of Nursing, Mansoura University, Mansoura City, Egypt

**Keywords:** Workplace spirituality, Self-efficacy, Job embeddedness, Nursing, Mediation analysis

## Abstract

**Introduction:**

Egypt’s healthcare system faces significant challenges for the nursing workforce, including high turnover rates and job dissatisfaction. Understanding the factors that enhance nurse retention and self-efficacy is crucial for improving healthcare delivery in the Egyptian context, where workplace spirituality may play a significant role, given cultural and religious values.

**Aim:**

This study examined the relationships between workplace spirituality, self-efficacy, and job embeddedness among nurses and investigated whether self-efficacy mediates the relationships among these factors.

**Methods:**

A cross-sectional study was conducted in 2025 with 305 nurses employed at the Oncology Center of Mansoura University Hospitals. Data were collected using the workplace spirituality scale, self-efficacy scale, and job embeddedness scale. Statistical analyses included Pearson correlation, multiple regression, hierarchical regression, and path analysis using SPSS 23.0 and AMOS 23.0.

**Results:**

Correlation analyses revealed the strongest relationship between workplace spirituality and job embedding (*r* = 0.365), moderate correlations between self-efficacy and job embeddedness (*r* = 0.250), and weak associations between workplace spirituality and self-efficacy (*r* = 0.166). Regression analyses confirmed workplace spirituality was a significant predictor of outcomes with different effect sizes. While workplace spirituality weakly predicted self-efficacy (β = 0.166, 2.8% variance), it strongly predicted job embeddedness (β = 0.365, 13.3% variance). Years of experience dominated the self-efficacy prediction (27.6% variance). Path analysis revealed self-efficacy as a partial mediator, although the mediation effect was modest (indirect effect = 0.032) compared to the direct effect (0.270).

**Conclusions:**

Workplace spirituality significantly predicted nursing self-efficacy and job embeddedness with more potent effects on job embeddedness. Self-efficacy partially mediates this relationship; however, workplace spirituality influences job embeddedness primarily through direct pathways, suggesting that multiple mechanisms enhance nurses’ retention and organizational attachment in nursing environments.

**Clinical trial number:**

Not applicable.

## Introduction

 The nursing profession has unparalleled difficulties in modern healthcare settings, with ongoing problems of turnover, burnout, and staffing shortages jeopardizing the stability of healthcare systems worldwide [1, 2]. Egypt’s healthcare system faces significant challenges for the nursing workforce, including high turnover rates, the intent to retain within its settings, and job dissatisfaction [3, 4]. As healthcare organizations seek sustainable solutions to retain skilled nursing professionals, researchers have increasingly focused on the psychological and spiritual factors that may enhance nurses’ commitment to their roles and organizations [5].

Workplace spirituality has emerged as a multifaceted concept that significantly affects various aspects of the nursing profession, including job performance, organizational commitment, and overall well-being [6]. A comprehensive model for improving workplace spirituality in nursing includes nursing education, organizational context, recruitment orientation, work environment experience, training programs, and harmonious working relationships [7]. Workplace spirituality is characterized by a feeling of connectivity and congruence with company principles, offering a profound sense of purpose and significance in professional duties, including aspects such as inner life, meaningful work, and community [8, 9]. Research indicates that workplace spirituality enhances nurses’ job performance by cultivating feelings of inner life, meaningful work, and community, which are essential for integrating personal and professional values and improving job satisfaction [8]. While workplace spirituality, self-efficacy, and job embeddedness have been studied individually in various organizational contexts, little is known about their interrelationship and predictive influence on nurse retention, particularly in Egypt. Specifically, the mechanisms through which workplace spirituality may influence job embeddedness, and whether self-efficacy mediates this relationship, remain underexplored. This gap represents a significant limitation in our understanding of the psychological and spiritual factors that contribute to nurse retention in healthcare settings.

Nurses’ self-efficacy is the belief in their ability to execute tasks and manage prospective situations [10]. Self-efficacy is crucial for nurses, especially in environments characterized by frequent changes and high demands, as it enhances their confidence in their ability to handle change, thereby reducing turnover intention and promoting retention [11]. Research indicates that nurses with greater organizational embeddedness report greater perceived self-efficacy, while self-efficacy allows nurses to enhance their confidence, fulfill their skills, and improve their expertise [11]. Given the complex and often unpredictable nature of healthcare environments, nurses with higher self-efficacy are better equipped to navigate clinical uncertainties and provide quality patient care, while maintaining their psychological well-being [12].

Compared with traditional job satisfaction measures, job embeddedness, conceptualized as how employees feel connected to their jobs and organizations, has gained recognition as a more comprehensive predictor of turnover intention [13]. The job embeddedness theory comprises two dimensions (organizational and community) and three constructs: fit-to-role, indicating the employee’s compatibility with the organization and community; links, which connect the individual to the organization through resources and collaborative decision-making; and sacrifices upon departure, reflecting the employee’s awareness of the losses incurred by leaving the position [14, 15]. Job embeddedness is crucial for retention, as it cultivates a feeling of belonging and commitment, with new meta-analytic results indicating a modest negative connection between job embeddedness and turnover intention [16].

The interplay between self-efficacy and job embeddedness is evident, as self-efficacy mediates the relationship between organizational support and job embeddedness, suggesting that enhancing self-efficacy can strengthen job attachment [17]. Moreover, self-efficacy and job embeddedness are inversely related to burnout, with higher levels leading to reduced burnout among nurses [18]. Research has demonstrated that self-efficacy positively influences job embeddedness, suggesting that nurses who believe in their capabilities are more likely to feel integrated and committed to their workplace [19]. Additionally, self-efficacy directly and indirectly affects self-leadership and job embeddedness [20].

Comprehending these linkages is essential for identifying causal pathways among these variables, which may guide focused therapies that address many factors concurrently, instead of addressing them in isolation. Considering the moral and spiritual aspects intrinsic to the nursing profession, enhancing support for nurses and facilitating their discovery of a spiritual base through mindful activities that evoke pleasant emotions may significantly improve their optimism and job satisfaction. The incorporation of these principles into nursing education and management practices may enhance nurse retention and work satisfaction, thus benefitting healthcare organizations by lowering expenses related to high turnover rates [21]. These findings may inform evidence-based approaches to creating work environments that support nurses’ spiritual well-being, while enhancing their professional confidence and organizational commitment, as research indicates that job satisfaction serves as an explanatory variable for nurse retention.

### Theoretical framework and conceptual relations

The connections between spirituality at work, self-efficacy, and job embeddedness have theoretical bases describing their psychological and social processes. Based on meaning-making theory [22] and self-determination theory [23], workplace spirituality affects self-efficacy through several pathways. The spiritual significance in nurses’ work makes them psychologically confident that their work contributes to healing and care. The meaning-making process transforms daily activities into meaningful ones, promoting nurses’ belief in their ability to complete duties [24, 25].

Spiritual workplace experiences, marked by compassion and supportive relationships, provide emotional resources that promote resilience. This enables nurses to view difficult situations as manageable, increasing self-efficacy beliefs [26]. Through perceived organizational support [27], workplace spirituality shows employees that the organization values their well-being, creating a climate of safety and trust that builds confidence [28].

Job embeddedness theory [29], states that employees stay due to fit, links, and sacrifice. Spiritual organizational experiences enhance person-organization fit by aligning with personal values and organizational culture [30]. When nurses feel their workplace respects spiritual values, their embeddedness increases through alignment with organizational identity [31]. Workplace spirituality strengthens social connections through relationships based on shared values, forming bonds that integrate nurses into the organization [32, 33].

Third, a spiritually meaningful workplace represents an important sacrifice as employees would lose both employment and a means of purpose and value-based community. This perceived loss increases turnover’s psychological cost and job embeddedness. Self-Efficacy as a mediator: psychological empowerment pathway theoretically, self-efficacy mediates the connection between spirituality and job embeddedness through psychological empowerment processes [34, 35]. While spirituality directly relates to job embeddedness through value coherence and social bonding, it also indirectly connects via self-efficacy. As workplace spirituality increases nurses’ confidence in their skills, their enhanced self-efficacy increases their sense of competence, autonomy and influence - key aspects of psychological empowerment [31, 36]. Empowered employees invest more in organizational functions, build better coworker relationships, and feel more fitting to the organization, enhancing job embeddedness [36]. The mediation is partial since workplace spirituality can influence job embeddedness through non-psychological factors like value congruence and social bonds [6]. This theoretical view suggests workplace spirituality impacts job embeddedness both directly through organizational processes and indirectly via psychological processes, including self-efficacy.

This study adopts an integrated theoretical approach to investigate the influence of workplace spirituality on nurses’ psychological functioning and organizational attachment. It addresses a notable gap in literature, as previous research has often examined these constructs in isolation rather than within a cohesive framework. The study aims to evaluate how workplace spirituality affects nurses’ self-efficacy and job embeddedness in Egyptian healthcare settings and to ascertain whether self-efficacy mediates the relationship between workplace spirituality and job embeddedness.

Understanding these relationships is vital for identifying causal pathways that can guide targeted interventions addressing multiple factors simultaneously rather than individually. Given the moral and spiritual dimensions inherent in nursing, enhancing support through mindful activities that evoke positive emotions may significantly boost optimism and job satisfaction. Integrating these principles into nursing education and management practices could improve nurse retention and job satisfaction, ultimately benefiting healthcare organizations by reducing expenses related to high turnover rates. From a practical standpoint, identifying these relationships can inform the development of evidence-based interventions that reduce turnover and burnout, potentially lowering the substantial costs associated with nurse recruitment and training while enhancing patient care quality. From a scientific perspective, this study contributes to theoretical models by incorporating workplace spirituality as a predictor within the psychological and organizational framework of nurse retention, advancing the understanding of the causal pathways through which spiritual workplace experiences influence career decisions and organizational commitment.

This study advances three key scientific conversations: organizational psychology in nursing by examining the integration of spiritual and psychological factors in retention models, workplace spirituality research by demonstrating its predictive role in job embeddedness, and occupational health literature by elucidating mediation mechanisms explaining how spiritual workplace experiences translate into organizational attachment. These covering these theoretical gaps, the findings will inform evidence-based approaches to creating work environments that support nurses’ spiritual well-being while enhancing professional confidence and organizational commitment, ultimately advancing the understanding of multidimensional factors influencing nurse retention in healthcare organizations.

## Methods

### Research questions

1. How does workplace spirituality influence nurses’ self-efficacy within healthcare settings?
2. What is the relationship between workplace spirituality and job embeddedness among nurses?
3. Does workplace spirituality significantly correlate with nursing self-efficacy and job embeddedness?

Question 3 has been integrated as a foundational hypothesis supporting Questions 1 and 2, establishing a conceptual link among workplace spirituality, self-efficacy, and job embeddedness.

### Methods

#### Study design and setting

This study employed a cross-sectional design, which is appropriate for examining associations and predictive relationships between variables [37]. The study was conducted at the department of oncology centers between April 30 and May 30, 2025. The center, which is in Mansoura City after Mansoura University Hospitals, Dakahelia Governorate, represents the Ministry of Higher Education. It could accommodate up to 550 beds. Medical, surgical, hematological, pediatric, intensive care unit, surgical, and outpatient services are among the many high-quality healthcare services offered by the Oncology Center to satisfy the needs of the Delta Region.

#### Participants, sample size, and sampling

The convenience sample was comprised of 305 staff nurses who provided patient care to all departments. The participants had to be staff nurses at the Oncology Center (OC) during data collection, have at least one year of experience across both genders, and provide written informed consent. The participants also had to work in their nursing post for six months. Nurses who were on leave during the study, those with less than one year of current employment, and those who refused or failed to provide informed consent were excluded.

According to the data obtained from the nursing director’s office at the Oncology Center, 450 nurses were available at the time of data collection. *Using G* Power 3.1.9.7 software, with an anticipated medium effect size (f² = 0.15), a statistical power of 0.90, an alpha level of 0.05, and accounting for five predictor variables representing the dimensions of workplace spirituality, the minimum required sample size was calculated to be 115 participants. However, recognizing the demanding work environment of oncology nurses at Mansoura University Hospitals and the potential challenges in data collection, including fatigue, shift work schedules, and incomplete responses, the researchers targeted a substantially larger convenience sample of 305 nurses.

#### Study measurements

The study’s data were gathered via three specific measures, including the Nurses’ Demographic and Clinical Data Questionnaire, which explicitly focuses on age and years of experience. The Workplace Spirituality Scale was selected to directly answer RQ1 on the impact of workplace spirituality on self-efficacy and RQ2 on its impact on job embeddedness. To answer the question (RQ1) whether nurses are confident in their abilities, and to investigate whether it may be a mediator (RQ3), the General Self-Efficacy Scale was picked. The Job Embeddedness Scale was applied to the level of nurse perception of being embedded in the organizational context, which answers RQ2 and RQ3 about the predictive relationships and mediation effects. All the assessments were conducted in Arabic.

1. **Workplace Spirituality Scale**

WSS was developed by Pradhan et al. [38]. This scale was used to assess the spirituality of nurses in the workplace. The instrument has 30 statements categorized into four variables: spiritual orientation (12 statements), compassion (four statements), meaningful job (eight statements), and alignment of values (six statements). Staff nurses’ views were assessed using a five-point Likert scale with options ranging from (1) strongly disagree to (5) strongly agree. 1 = strongly disagree, 2 = disagree, 3 = neutral, 4 = agree, 5 = strongly agree.

2. **Self-Efficacy Scale**

Sherer et al. [39] created it to assess self-efficacy in nurses. The measure has 23 items categorized into two subscales: the general self-efficacy subscale, which includes 17 questions, and the social self-efficacy subscale, which consists of 6 items. Responses were quantified on a 5-point Likert scale ranging from 1 to 5 (1 = strongly disagree, 2 = disagree, 3 = neutral, 4 = agree, and 5 = strongly agree).

3. **Job Embeddedness Scale**

Mitchell et al. [29] developed it to evaluate nurses’ job embeddedness inside their workplace. It has 23 questions classified into three subscales: organizational affiliation (seven items), organizational fit (seven items), and organization-related sacrifice (nine items). The answers were evaluated via a 5-point Likert scale ranging from strongly disagree (1) to strongly agree (5).

#### Pilot study

Prior to data collection, a pilot study involving 10% (*n* = 30) of the nursing personnel across various departments at the Oncology Center, comprising 25 nurses and five head nurses, was conducted, leading to minor modifications in wording to enhance clarity for the target population. This pilot study was conducted to assess the completeness, applicability, clarity, and usefulness of the tools, and to estimate the time needed to complete the questionnaire. No items were added or removed, and the pilot sample was excluded from the main study. The main study sample excludes the pilot research sample.

#### Validity and reliability

The Workplace Spirituality, Self-Efficacy, and Job Embeddedness Scale were meticulously translated into Arabic. Experts proficient in English and Arabic were engaged to guarantee that the translations maintained their accuracy and cultural relevance. Two bilingual research team members carried out the initial translations. Two independent bilingual experts who were not affiliated with the research team subsequently back-translated these translations into English to ensure linguistic consistency and to address discrepancies. Following the translation process, each instrument’s face validity assessment was conducted by incorporating feedback from seven experts in nursing sciences. The experts provided feedback encompassing recommendations for rephrasing specific items to improve clarity and to guarantee that the target population would readily comprehend the terminology. Considering this feedback, slight modifications were implemented to enhance the clarity and relevance of questionnaire items.

Arabic versions of the items were assessed using the Content Validity Index (CVI). The experts rated each issue from one (not relevant) to four (extremely relevant). The ratio of experts rating an item as three or four to the total number of experts in the evaluation determines the item-level content validity index (I-CVI). The item-level content validity indices were averaged to obtain the scale-level content validity index (S-CVI). CVI values of 0.92 for the workplace spirituality scale, 0.89 for self-efficacy, and 0.90 for job embeddedness indicate strong content validity. The workplace spirituality, self-efficacy, and job embeddedness scales had Cronbach’s alpha values of 0.91, 0.88, and 0.92, respectively. The data indicated excellent instrument dependability.

**Fig. 1:**
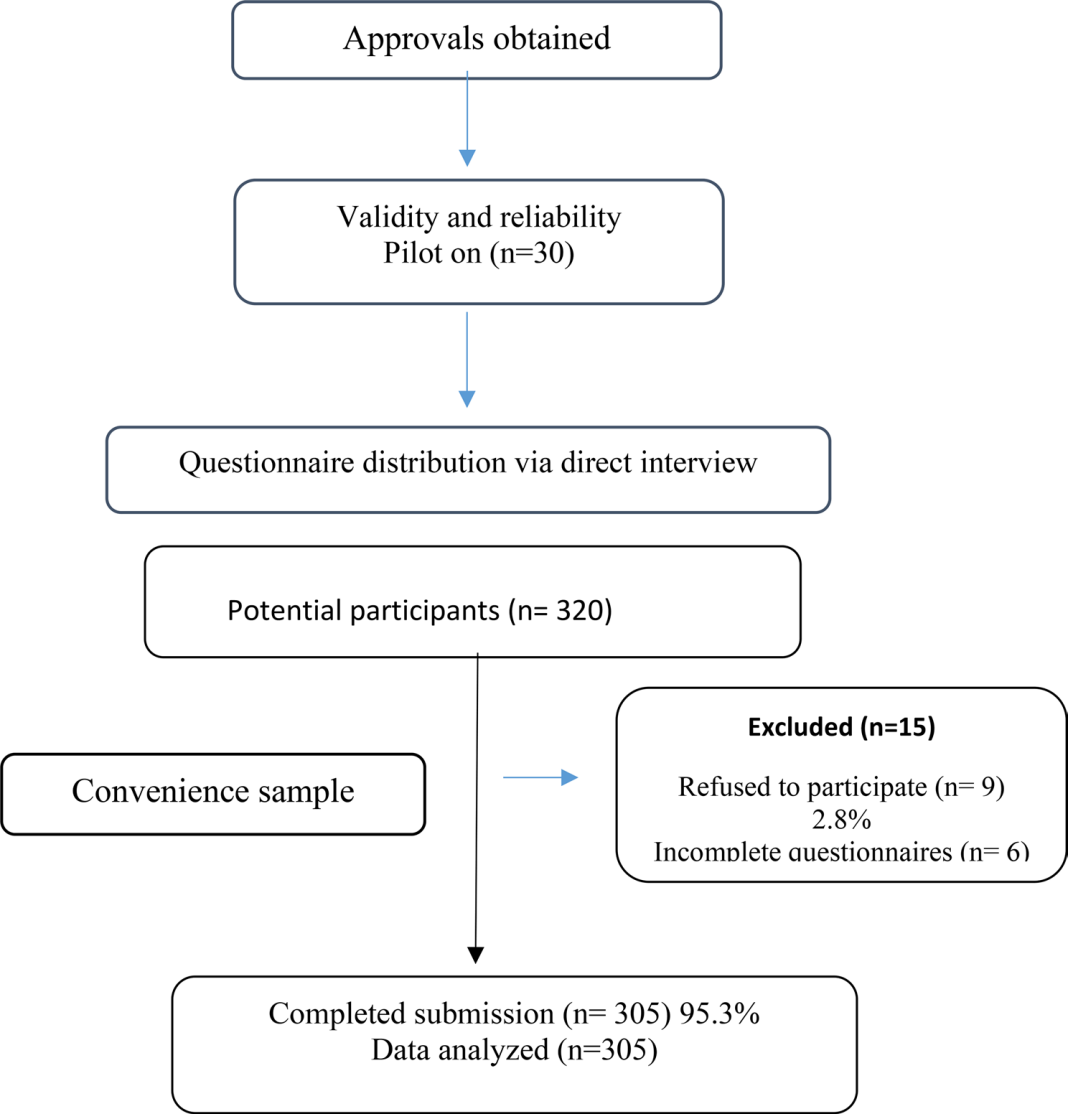
Flow chart of nurse enrollment

### Data collection

Authorization to conduct the study was obtained from the hospital director and nursing administration, and ethical approval was granted by the Mansoura University Faculty of Nursing Research Ethics Committee. The data were collected from April to May 2025. Prior to data collection, the research team coordinated with the head nurses in each ward to explain the study’s purpose and obtain permission for participant recruitment. After approval, paper-based questionnaires were distributed by the research team to eligible nurses during break times to minimize disruption of patient care. Participants completed the questionnaires individually in designated quiet areas in their respective units.

Each questionnaire was anonymous and no information was collected. The completed questionnaires were placed in sealed envelopes and immediately retrieved by the research team to ensure confidentiality. The opening page of the questionnaire included detailed information regarding the study objectives, data usage, and confidentiality assurance. Participation was voluntary and written informed consent was obtained before the completion of the study.

To ensure accuracy, researchers conducted brief structured interviews with participants during breaks, confirming completeness and clarifying responses, as needed. Each questionnaire required approximately 10–15 min to complete. Of the 320 nurses invited, six (1.9%) submitted incomplete surveys and nine (2.8%) declined participation, resulting in a total of 305 valid questionnaires and a 4.7% attrition rate. The data collection process is illustrated in Fig. 1.

#### Handling attritions and biasing

To minimize attrition, the researchers maintained close communication with head nurses and nursing directors to facilitate nurse participation during different shifts. Questionnaires were distributed and collected in person by the research team, ensuring immediate follow-up on incomplete responses. Consequently, the attrition rate was low (4.7%). Six nurses (1.9%) submitted incomplete questionnaires, and nine (2.8%) declined participation. The characteristics of non-respondents were compared with those of respondents, and no significant differences were found in demographic variables (age, years of experience, department), indicating a limited attrition bias.

Although convenience sampling was employed, recruitment spanned all departments and was adjusted to enhance representativeness within the center. The distribution of clinical departments and shift systems mirrors the typical staffing structures in Egyptian healthcare facilities, affirming the representativeness of our sample and the potential for the findings to be generalized to other settings with similar conditions. To mitigate potential response and selection biases, participation was voluntary, anonymity and confidentiality were assured, and participants were informed that their responses would not impact their professional standing. Additionally, data collectors were trained to maintain a neutral stance and clarify ambiguous items without influencing participant responses. These measures ensured the validity and reliability of the data.

#### Statistical analysis

The IBM SPSS software package version 23.0 was used to analyze the data fed into the computer. The data distribution normality was tested with Kolmogorov-Smirnov and Shapiro-Wilk tests and by histograms. Findings showed that the key study variables were relatively normally distributed (*p* > 0.05 all normality tests). Pearson correlation coefficients were suitable to determine the bivariate relationships between workplace spirituality, self-efficacy, and job embeddedness. To determine the direct and indirect effects of workplace spirituality on job embeddedness, which are mediated by self-efficacy, path analysis was performed using AMOS 23.0. Path analysis using AMOS has been chosen as a mediation test because of a number of strengths: (1) it estimates all the paths in the mediation model simultaneously, offering more precise standard errors than sequential regression methods, (2) it has extensive model fit measures that can be used to evaluate the extent to which the theoretical model fits the observed data, (3) it can test the direct and indirect effects of the model within a single analysis, and (4) AMOS is also commonly used in nursing research, and it makes it easy to compare it with other studies of the same type. The significance of the results was assessed at the 5% level.

### Analytical strategy

#### The following analytical strategy was employed to address the research questions

**Fig. 2:**
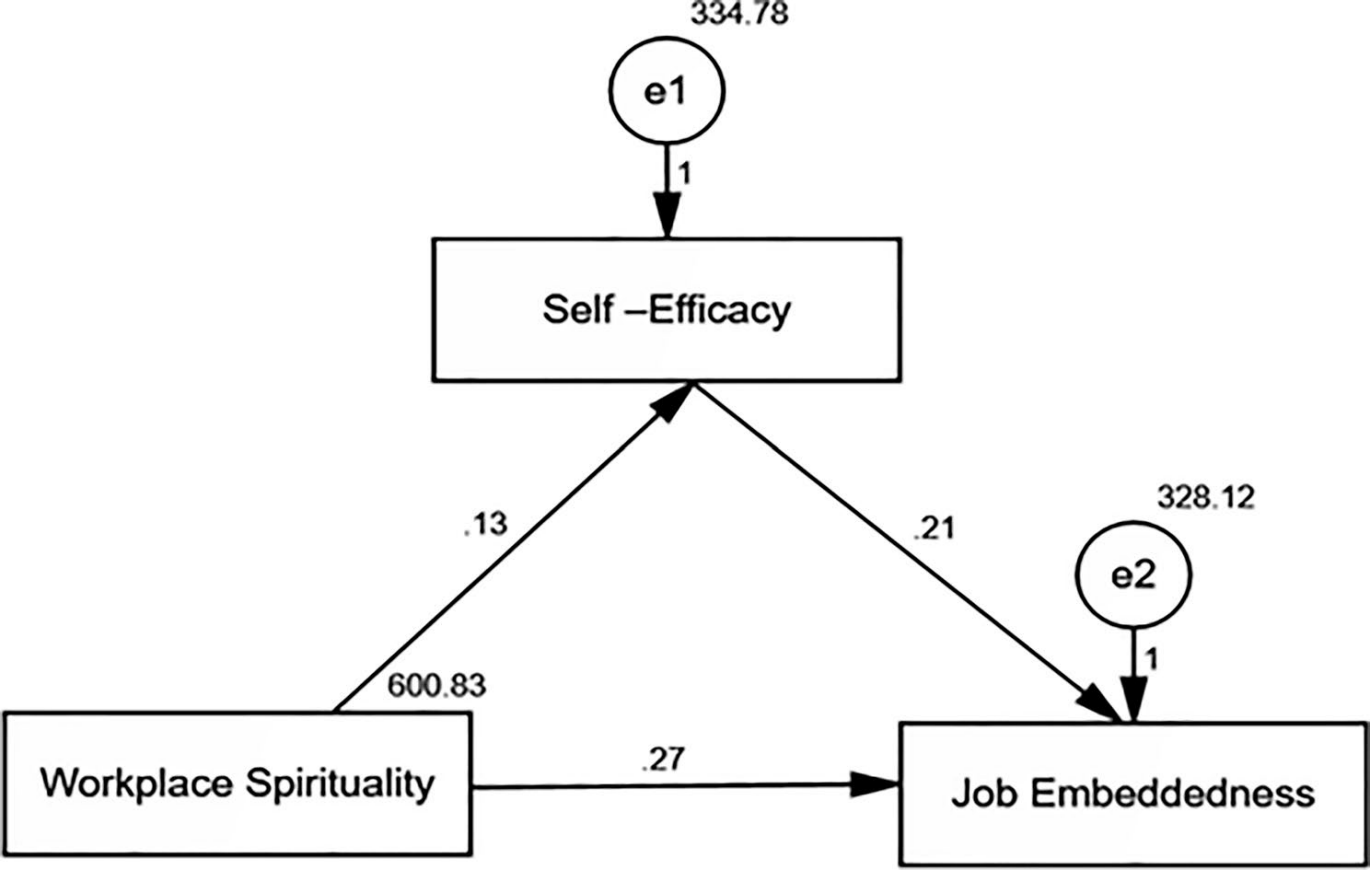
Path analysis showing the direct and indirect effects of workplace spirituality on job embeddedness mediated by self-efficacy. Model fit parameters CFI; IFI; RMSEA (1.000; 1.000; 0.096). CFI = comparative fit index; IFI = incremental fit index; RMSEA = root mean square error of approximation. Model significance 21.750^*^(< 0.001^*^)

1. Research Question 1 (workplace spirituality predicting self-efficacy): Pearson correlation analysis (Table 3) was used to examine the relationship, followed by simple regression analysis (Table 4) to determine predictive power, and hierarchical regression (Table 6) to control for demographic factors.
2. Research Question 2 (workplace spirituality and job embeddedness): Similar analytical progression using Tables 3, 5, and 7.
3. Research Question 3 (mediation by self-efficacy): Path analysis (Fig. 2; Table 8) was used to examine the direct and indirect effects.

**Table 1:** Demographic data (*n* = 305)

Demographic data	No	%
**Age**		
20–29	161	52.8
30–39	121	39.7
≥ 40	23	7.5
**Mean ± SD**	**29.9 ± 6.3**	
**Years of experience**		
< 5	43	14.1
5 - <10	81	26.6
10 - <15	61	20.0
15 - <20	81	26.6
≥ 20	39	12.8
**Mean ± SD**	**12.0 ± 6.4**	

**Table 2:** Descriptive analysis of the study variable workplace spirituality, self–efficacy, and job embeddedness scales scores

Study variables	Total score		Percent score	
	Mean	±SD	Mean	±SD
**Workplace Spirituality scale**	128.67	24.55	81.51	21.16
Spiritual Orientation	53.07	8.81	85.55	18.35
Compassion	16.98	3.65	81.13	22.80
Meaningful Work	33.50	7.17	79.70	22.42
Alignment of Values	25.12	5.61	79.67	23.40
**Self–Efficacy Scale**	**100.14**	**18.58**	**81.92**	**20.90**
Factor 1: General Self-Efficacy	75.45	13.51	85.95	19.87
Factor 2: Social Self-Efficacy	24.69	5.62	77.88	23.40
**Job Embeddedness scale**	**101.44**	**19.92**	**85.55**	**21.55**
Links to Organization	31.78	6.38	88.51	22.80
Fit to Organization	31.07	6.12	85.96	21.87
Organization-Related Sacrifice	38.58	8.52	82.18	23.65

**Table 3:** Correlations between the studied variables (*n* = 305)

		Workplace Spirituality	Self –Efficacy	Job Embeddedness
Workplace Spirituality	r			
	p			
Self –Efficacy	r	0.166*		
	p	0.004*		
Job Embeddedness	r	0.365*	0.250*	
	p	< 0.001*	< 0.001*	

r: Pearson coefficient

*: Statistically significant at *p* ≤ 0.05

**Table 4:** Multiple regression to determine the predictive power of workplace spirituality for self-efficacy (*n* = 305)

Variable	B	Beta	t	*p*	95% CI	
					LL	UL
Workplace Spirituality	0.126	0.166	2.930*	0.004*	0.041	0.210
R^2^=0.028, Adjusted R^2^ = 0. 024, F = 8.586^*^, *p* < 0.001^*^						

F, p: f and p values for the model; R^2^: Coefficient of determination; B: Unstandardized Coefficients; Beta: Standardized Coefficients; t: t test of significance LL: lower limit; UL: upper limit; *: Statistically significant at *p* ≤ 0.05

**Table 5:** Multiple regression to determine the predictive power of workplace spirituality for job embeddedness (*n* = 305)

Variable	B	Beta	t	*p*	95% CI	
					LL	UL
Workplace Spirituality	0.296	0.365	6.827*	< 0.001*	0.211	0.382
R^2^=0.133, Adjusted R^2^ = 0. 130, F = 46.6410^*^, *p* < 0.001^*^						

F, p: f and p values for the model; R^2^: Coefficient of determination; B: Unstandardized Coefficients; Beta: Standardized Coefficients; t: t test of significance; LL: lower limit UL: upper limit; *: Statistically significant at *p* ≤ 0.05

**Table 6:** Hierarchical regression to control the effects of the demographic variables of workplace spirituality on self-efficacy (*n* = 305)

Variable	B	Beta	t	*p*	95% CI		
					LL		UL
Step1							
Years of experience	1.526	0.526	10.752*	< 0.001*	1.247		1.805
R^2^=0.276, Adjusted R^2^ = 0. 274, F = 115.600^*^, *p* < 0.001^*^							
Step2							
Years of experience	1.524	0.525	10.919*	< 0.001*	1.249		1.798
Workplace Spirituality	0.124	0.163	3.397*	0.001*	0.052		0.195
R^2^=0.303, Adjusted R^2^ = 0. 298, F = 65.580^*^, *p* < 0.001^*^							
**Excluded Variable**	**Beta in**	**t**	**Sig.**	**Partial Correlation**	**Collinearity Statistics**		
Age	0.038	0.771	0.441	0.044	0.936		

F, p: f and p values for the model; R^2^: Coefficient of determination; B: Unstandardized Coefficients Beta: Standardized Coefficients; t: t test of significance; LL: lower limit; UL: upper limit; *: Statistically significant at *p* ≤ 0.05

**Table 7:** Hierarchical regression to control for the effects of the demographic variables of workplace spirituality on job embeddedness (*n* = 305)

Variable		B		Beta	t	*p*	95% CI	
							LL	UL
Step1								
Workplace Spirituality		0.296		0.365	6.827*	< 0.001*	0.211	0.382
R^2^=0.133, Adjusted R^2^ = 0. 130, F = 46.610^*^, *p* < 0.001^*^								
Step2								
Workplace Spirituality		0.289		0.356	6.929*	< 0.001*	0.207	0.371
Age		0.844		0.267	5.191*	< 0.001*	0.524	1.164
R^2^=0.24, Adjusted R^2^ = 0. 199, F = 38.776^*^, *p* < 0.001^*^								
**Excluded Variable**	**Beta in**		**t**		**Sig.**	**Partial Correlation**	**Collinearity Statistics**	
**Years of experience**	0.086		1.620		0.106	0.093	0.937	

F, p: f and p values for the model; R^2^: Coefficient of determination; B: Unstandardized Coefficients; Beta: Standardized Coefficients; t: t test of significance; LL: lower limit; UL: upper limit; *: Statistically significant at *p* ≤ 0.05

**Table 8:** Direct and indirect effects

Variable 1		Variable 2	Direct effect	Indirect effect	C.*R*	*p* value
Self-Efficacy	←	Workplace Spirituality	0.126		2.935*	0.003*
Job Embeddedness	←	Workplace Spirituality	0.270	0.032	6.281*	< 0.001*
Job Embeddedness	←	Self-Efficacy	0.209		3.681*	< 0.001*

## Results

### Demographic characteristics

The study participants included 305 nurses with a predominantly young workforce; over half (52.8%) were aged 20–29 years, and nearly 40% were in their thirties, resulting in a mean age of 29.9 years. The experience distribution was more varied, with participants having an average of 12.0 years of experience, although the largest groups had either 5–10 or 15–20 years of experience (26.6% each) (Table 1).

### Descriptive analysis of workplace spirituality, self–efficacy, and job embeddedness scales scores

Table 2 reveals that generally high mean scores across all study variables, indicating that nurses in the oncology center reported strong workplace spirituality, self-efficacy, and job embeddedness. Among the workplace spirituality dimensions, spiritual orientation demonstrated the highest mean percentage score (85.55%), suggesting that nurses strongly valued purpose and connection in their work. In contrast, alignment of values and meaningful work showed slightly lower mean percentage scores (79.67% and 79.70%, respectively), reflecting moderate variability in how nurses perceived congruence between personal and organizational values.

General self-efficacy scored higher (85.95%) than social self-efficacy (77.88%), indicating that nurses tend to feel more confident in handling professional tasks than in social interactions within their workplace. Similarly, for job embeddedness, links to the organization scored highest (88.51%), followed by fit to the organization (85.96%) and organization-related sacrifice (82.18%), suggesting that nurses are strongly connected to their workplace through relationships and compatibility with organizational culture, while attachment based on potential loss or sacrifice was slightly lower (Table 2).

### Correlation patterns and relationships

As shown in Table 3, the WPS demonstrated a positive and statistically significant correlation with both self-efficacy (*r* = 0.166, *p* = 0.004) and job embeddedness (*r* = 0.365, *p* < 0.001). This suggests that nurses who experienced a stronger sense of spirituality at work tended to report higher self-efficacy and a greater sense of attachment to their organization. Similarly, self-efficacy showed a moderate positive correlation with job embeddedness (*r* = 0.250, *p* < 0.001), indicating that nurses who felt more confident in their professional abilities were more likely to remain engaged and committed to their workplace.

These results reveal a consistent pattern of mutually reinforcing relationships between the three variables. Although the correlation between workplace spirituality and self-efficacy was relatively modest compared to that between workplace spirituality and job embeddedness, all associations were positive and significant, underscoring the interdependence between nurses’ psychological resources and organizational connectedness.

### Predictive relationships and variance explanation

Multiple regression analysis was conducted to determine whether workplace spirituality predicted nurses’ self-efficacy (Tables 4 and 5). As shown in Table 4, the WPS was found to be a significant positive predictor of self-efficacy (β = 0.166, *p* = 0.004), explaining approximately 2.8% of the variance in self-efficacy scores (R² = 0.028). This finding indicates that nurses who perceive higher levels of spirituality in their workplace tend to report greater confidence in their professional abilities. Although the overall variance explained was modest, the significant association highlights the meaningful influence of spiritual work environments on nurses’ psychological empowerment.

As shown in Table 5, workplace spirituality significantly predicted job embeddedness (β = 0.365, *p* < 0.001), accounting for 13.3% of the variance (R² = 0.133). This result demonstrates that higher perceptions of workplace spirituality are strongly associated with greater attachment and commitment to the organization. Compared to its effect on self-efficacy, workplace spirituality exhibited stronger predictive power for job embeddedness, suggesting that a spiritually supportive environment **is positively related to** nurses’ sense of belonging and organizational connection more directly than their self-confidence alone.

### Demographic control and hierarchical regression effects

Hierarchical regression analysis was performed to examine the influence of workplace spirituality on self-efficacy, while controlling demographic varivariables (es 6 and 7). As shown in Table 6, in step 1, years of experience significantly predicted self-efficacy (β = 0.526, *p* < 0.001), explaining 27.6% of the variance (R² = 0.276). In Step 2, after introducing workplace spirituality into the model, both years of experience (β = 0.525, *p* < 0.001) and workplace spirituality (β = 0.163, *p* = 0.001) remained significant predictors. The model explained 30.3% of the variance in self-efficacy (R² = 0.303), indicating that workplace spirituality contributes to nurses’ self-efficacy, even after accounting for professional experience.

Age was not a significant predictor (*p* = 0.441), suggesting that self-efficacy is more strongly associated with work experience and workplace spirituality than chronological age. These findings highlight that exposure to spiritually supportive work environments may enhance nurses’ confidence in their professional skills.

As shown in Table 7, the WPS was a significant predictor of job embeddedness in Step 1 (β = 0.365, *p* < 0.001), accounting for 13.3% of the variance (R² = 0.133). When age was added in Step 2, both workplace spirituality (β = 0.356, *p* < 0.001) and age (β = 0.267, *p* < 0.001) remained significant predictors, increasing the model’s explanatory power to 24% (R² = 0.240). This indicates that both higher WPS scores and older age are associated with stronger job embeddedness among nurses.

Years of experience were excluded as a non-significant predictor (*p* = 0.106*), implying that age and workplace spirituality exert a greater influence on nurses’ retention and organizational attachment than total work experience alone. Together, these results reveal that nurses who perceive their work environment as spiritually supportive and older are more likely to be embedded within their organizations.

### Mediation analysis and path relationships

Figure 2 demonstrates the direct and indirect effects of workplace spirituality on job embeddedness mediated by self-efficacy, showing that workplace spirituality positively influences self-efficacy, which, in turn, is positively related to job embeddedness. The model fit indices (CFI = 1.000, IFI = 1.000, RMSEA = 0.096) confirmed a good fit, supporting the appropriateness of the specified model. The increased RMSEA can be an indication of the complexity of any model or sample-specific peculiarities. Nonetheless, considering other metrics of fit were within plausible levels and the theoretical and empirical evidence of the existing relationships as posited in the hypothesis, the model was considered satisfactory in testing the mediation hypothesis.

Additionally, Table 8 presents both the direct and indirect effects, confirming that mediation through self-efficacy was statistically significant (indirect effect = 0.032, *p* < 0.001), accounting for only 10.6% of the total effect. The direct effect of workplace spirituality on job embeddedness (0.270) was notably stronger than the indirect effect through self-efficacy (0.032), suggesting that, while self-efficacy contributes to the relationship, workplace spirituality primarily affects job embeddedness through other mechanisms. This result demonstrates that future studies are required to investigate other mediating mechanisms. The total effect (0.302) indicated that workplace spirituality was positively related to employee embeddedness via multiple pathways, with the self-efficacy pathway representing one significant but limited component of this broader relationship (Table 8).

## Discussion

Nurses experience a profound connection with their colleagues and the organization, alongside a harmonious alignment between their values and those upheld by the institution. They experienced a profound sense of satisfaction with their work. These factors suggest that individuals who feel integrated within the work system are inclined to remain in their positions, as these roles offer them meaningful engagement that aligns with their values and beliefs. This research explored the relationship between workplace spirituality and its influence on nursing self-efficacy and job embeddedness within the context of an oncology center.

The present results revealed that workplace spirituality was well above the average (81.51%) of the total possible score. This result might be due to nursing managers dealing with staff nurses with effective communication, and staff nurses being supported by their managers; they work in a positive environment and have development opportunities. Similarly, Mohamed et al. [40], who studied engagement, work-spirituality, and thriving, reported that most nurses had a moderate level of workplace spirituality. Additionally, this result is consistent with that of Khalaaf et al. [41], who studied workplace spirituality and its influence on staff nurses’ motivation and team effectiveness and reported that most nurses had a moderate level of workplace spirituality.

Our findings revealed that self-efficacy was high at 81.92% of the possible score, although general self-efficacy (85.95%) outperformed social self-efficacy (77.88%). These findings agree with previously published results. For example, Elsherif and Sabra [42] reported that approximately three-quarters of the studied nurses had moderate-to-high self-efficacy. Similarly, a study by Abdel-Azeem et al. [43] revealed that approximately two-thirds of staff nurses in the sample had moderate to high self-efficacy.

The results revealed that job embeddedness demonstrated the highest overall score at 85.55% of the total, with links to the organization being particularly strong at 88.51%, suggesting that employees felt well connected to their workplace. This may be because staff nurses have a network of positive relationships with coworkers who respect one another, as well as with head nurses and supervisors. They are inspired to perform the best of their abilities because they feel valued for their work. Through training programs offered by the organization that use a variety of methods, nurses also discovered that there is a great deal for them to grow both professionally and personally, which in turn encourages job embeddedness. Nurses are more likely to integrate into their positions in an atmosphere in which their beliefs, objectives, and priorities match their expectations regarding equity and recognize and promote a supportive workplace and professional autonomy.

These findings align with those of Ali et al. [44], who indicated that organizational factors, including job satisfaction, well-being, and salary, influence a moderate partial level of job embeddedness. Additionally, nurses’ psychological state serves as a predictive variable in examining the relationship between individual psychological resources and job embeddedness. This finding aligns with the work of Elsabahy et al. [45], who discovered that nearly half of nurses exhibited moderate job embeddedness. They found that a strong alignment in values presented the most significant potential for nurses to integrate deeply into their roles.

Conversely, Mahmoud and Obied [46] revealed that a diminished level of overall job embeddedness could be attributed to various factors, including insufficient income for nurses, especially amidst rising costs, and adverse working conditions stemming from restrictive policies and systems within university hospitals such as the inability to transition to another work environment without replacement.

While our findings align with prior research, important methodological differences warrant discussion. Unlike mohamed et al. [40], who sampled exclusively from academic teaching hospitals, our study included both academic and general hospitals, capturing greater workplace diversity. Elsherif and sabra [42] examined self-efficacy in intensive care units specifically, a high acuity setting that may amplify efficacy importance, whereas our broader hospital sample reflects more varied work contexts. Furthermore, our multidimensional measurement of workplace spirituality including meaningful work, sense of community, alignment with organizational values, and compassion provides more nuanced understanding compared to single composite scores used in some previous studies. Our use of the job embeddedness scale rather than turnover intention as the primary outcome represents a theoretical advancement, capturing the web of attachments keeping nurses in their roles rather than merely dissatisfaction pushing them away.

Our findings indicate that the most robust bivariate connection was between workplace spirituality and job embeddedness (*r* = 0.365). This suggests that people with heightened spiritual workplace experience are more likely to be embedded in their employment. Workplace spirituality was a much greater predictor of job embeddedness (β = 0.365, *p* < 0.001), indicating a considerable and practically significant effect size that underscores its importance in employee retention and attachment. This may be attributed to the ability of the WPS to foster a more enriching and supportive work environment for nurses, which is closely associated with improved patient and nurse results. Nurses who see their spiritual needs as recognized and fulfilled are more inclined to obtain significance and satisfaction from their careers. Workplace spirituality fosters feelings of purpose, belonging, and connection with business values, resulting in heightened employee engagement and dedication, subsequently enhancing work embeddedness.

The finding that workplace spirituality explained 13.3% of variance in job embeddedness represents a meaningful effect size with practical significance for retention interventions. Research on turnover costs suggests that each percent reduction in turnover can save hospitals substantial financial resources and protect patient care quality [47]. Thus, interventions targeting workplace spirituality may have meaningful impact on organizational outcomes, particularly given that the direct pathway from workplace spirituality to job embeddedness operates independently of self-efficacy, suggesting that creating spiritual workplace climates influences retention through multiple mechanisms simultaneously.

Multiple studies have corroborated similar results, including those of Jehanzeb et al. [48], who collected data from employees across different organizations in Pakistan. The findings indicate that workplace spirituality indirectly affects job embeddedness via mindfulness, suggesting that employees who perceive a sense of purpose are more engaged at work and exhibit enhanced emotional regulation, reinforcing their connections to their roles and organizations and increasing their likelihood of prolonged tenure. Jnaneswar and Sulphey [49] confirmed the beneficial association between workplace spirituality and job embeddedness. Similarly, Rajappan et al. [50] reported that employees who engage in workplace spirituality exhibit heightened attentiveness and presence, which increases the probability of developing a profound sense of embeddedness.

Steger and Dik [51] emphasized that employees dedicate substantial time to their job, rendering it a venue for goal pursuit, purpose exploration, and the cultivation of a feeling of belonging to colleagues and the business. The apparent feeling of loss upon leaving a company is more profound if individuals have established connections with their colleagues and the workplace. This incentivizes them to remain with the company and substantially decreases their turnover. To maintain employee engagement, firms should prioritize WPS enhancement of workplace spirituality.

The current findings indicate that self-efficacy was moderately associated with job embeddedness (*r* = 0.250) and less significantly associated with workplace spirituality (*r* = 0.166). The mediation study indicated that self-efficacy somewhat mediated the association between workplace spirituality and job embeddedness, although the effect size was small. This can be attributed to spiritually connected nurses exhibiting heightened engagement in their work, possessing a sense of purpose during challenging times and demonstrating greater confidence in their abilities. A spiritual workplace may also motivate employees to broaden their roles and assume additional responsibilities, potentially leading to enhanced self-efficacy and increased embeddedness.

In alignment with this conclusion, research has demonstrated that spirituality and self-efficacy are interconnected, both of which are significant motivators of work embeddedness [52]. Hsiao et al. [53] asserted that self-efficacy is not only an individual characteristic but also significantly linked to motivation and professional achievement. Spirituality enhances self-efficacy and serves as an effective coping mechanism against psychological challenges. Research has indicated a modest association between self-efficacy and spiritual well-being. These findings indicate that enhancing self-efficacy through spirituality may augment total work embeddedness.

Furthermore, Tunç et al. [54] reported that workers who view their lives as meaningful and purposeful are more inclined to esteem themselves and maintain confidence, even among challenges. This perspective reinforces individuals’ confidence in their talent and potential for success despite challenges, enabling them to see their lives as meaningful and worthwhile. The study results indicate that workers’ spirituality positively influences their self-efficacy.

The current findings align with those of Jehanzeb et al. [48], indicating that to enhance job embeddedness, organizations must focus on fostering a work environment that promotes spirituality, as its direct effect significantly surpasses its indirect influence via self-efficacy. This finding indicates that nurses’ job embeddedness relies more on a sense of connection, significance, and purpose at work than on enhancing their self-confidence. Rohini et al. [55] reported that self-efficacy beliefs might promote workplace spirituality, a crucial factor that has many sound effects. The knowledge-sharing atmosphere enhances this connection. The research demonstrated a robust correlation between intention to share knowledge, workplace spirituality, and self-efficacy.

In this regard, nurses with high self-efficacy, confidence in their abilities to succeed, a tendency to take control of their environment, and responsibility for their decisions experience organizational commitment and organizational justice. Involvement in organizational activities and a personal fit with organizational values align with the fit and link concepts of the job embeddedness theory [29].

Moreover, nurses perceived compatibility or comfort level with the organization and surrounding environment is also a crucial element in deciding their embeddedness. This means that individuals and organizations attempt to live their values more fully in their work (WPS). This calls for the link-minded attitudes of the individual and organization. Compatibility between the employee’s and the organization’s goals, personal values, as well as more immediate job-specific factors such as job knowledge, demands, skills, and abilities are the important factors that enable an employee to remain with the current job Lee et al. [56], and Mitchell et al. [29]suggests that the better the fit with the organization and the surrounding community, the stronger the ties with the organization.These deviations from western literature demonstrate that retention models from individualistic contexts need adjustment in collectivist settings. Conservation of resources theory’s focus on social resources may be more relevant in collectivist cultures, while social cognitive theory’s emphasis on individual efficacy may be less central. This cultural moderation of theoretical pathways warrants future cross-cultural research.

### Limitations

The limitations of this study include the representativeness of the sample, which consisted of hospital nurses, thereby potentially limiting the generalizability of the results to all nurses in Egypt or those working in university hospitals. Moreover, cross-sectional design was the primary limitation of this study. Our findings demonstrated significant associations and a mediation pattern consistent with our theoretical model, which cannot definitively establish that workplace spirituality causes changes in self-efficacy or job embeddedness. Convenience sampling may introduce selection bias, as nurses who chose to participate may differ systematically from those who declined.

Additionally, self-reported data collection may have led to memory bias. Moreover, reliance on hard copies may restrict the generalizability of the findings as it confines participation to particular care units and introduces logistical challenges. This method ensured the direct verification of respondents’ identities, thereby reducing the probability of duplicate responses. Despite these constraints, this study provides substantial evidence. Future research should investigate the effect of organizational variables on enhancing workplace spirituality and self-efficacy among nurses using a larger sample size.

### Nursing implications

This study has several advantages for enterprises. These findings highlight the significance of spirituality in the workplace and its relationship with increased job embeddedness. For organizational practice and leadership, organizations should prioritize workplace spirituality as a strategic retention intervention, as research has demonstrated that employees experiencing meaningful work aligned with personal values show significantly higher job embeddedness and lower turnover. Evidence-supported practices include value-aligned role design, regular recognition and feedback systems, cultural fit assessment during recruitment, and professional development opportunities. Based on this study’s findings, managers should implement value-driven leadership that actively cultivates workplace spirituality through transparent communication of organizational values, while human resources departments should integrate job embeddedness into talent management through ongoing fit assessments and retention initiatives that strengthen organizational connections.

Organizations should implement workplace spirituality initiatives targeting all four dimensions identified. This includes monthly reflection sessions where nurses discuss their work’s impact on patients, quarterly town halls where leadership demonstrates commitment to compassionate care through policies, peer support programs with mentorship meetings and team-building activities, and professional development opportunities that enhance self-efficacy while focusing on meaningful patient service. Organizations should conduct biannual surveys measuring workplace spirituality dimensions, self-efficacy, and job embeddedness to assess effectiveness, as workplace spirituality explained 13.3% of job embeddedness variance, suggesting programs could reduce turnover.

These recommendations operationalize the study’s theoretical framework. Meaningful work is implemented through reflection sessions and performance systems valuing compassion. Community sense is developed through peer support and mentorship fostering connections. Value alignment occurs through leadership communications demonstrating ethical commitment. Compassion is operationalized through protected time and recognition of compassionate behaviors. For Job Embeddedness Theory, fit improves through value-driven leadership, links strengthen through peer support, and sacrifice increases through spiritual investment. For Self-Efficacy Theory, interventions activate Bandura’s sources: mastery through workshops, vicarious learning through mentorship, social persuasion through recognition, and positive emotions through community support.

For nursing education, nursing curricula should incorporate job embeddedness content to address meaning in practice, value alignment, and professional community building, as the connection between embeddedness and nurse retention is well established. The present findings suggest that teaching future nurses to identify value-aligned workplace environments and cultivate spirituality in practice may support long-term retention and satisfaction, representing a novel educational approach that warrants further validation across diverse nursing contexts.

## Conclusion

The findings revealed that nurses reported the highest levels of job embeddedness, followed by self-efficacy, and moderate levels of workplace spirituality. Regression analyses showed that the WPS significantly predicted both self-efficacy and job embeddedness, with a stronger association observed for job embeddedness. Mediation analysis further indicated that self-efficacy partially mediated this relationship, suggesting that, while self-efficacy contributes to the link between workplace spirituality and job embeddedness, workplace spirituality primarily influences embeddedness through other mechanisms. Additionally, years of experience emerged as the strongest predictor of self-efficacy, while older nurses exhibited higher levels of job embeddedness.

## Data Availability

All the data are presented in the form of tables or figures. Additional data may be obtained from the corresponding author.

## Abbreviations

IRB: Institutional Review Board
